# The Utilization of Cultural Movements to Overcome Stigma in Narrative of Postnatal Depression

**DOI:** 10.3389/fpsyt.2020.532600

**Published:** 2020-10-30

**Authors:** Grace K. Elliott, Chris Millard, Ian Sabroe

**Affiliations:** ^1^Sheffield Medical School, University of Sheffield, Sheffield, United Kingdom; ^2^Lecturer in the History of Medicine and Medical Humanities, Department of History, University of Sheffield, Sheffield, United Kingdom; ^3^School of Health and Related Research, University of Sheffield, Sheffield, United Kingdom; ^4^Sheffield Teaching Hospitals National Health Service (NHS) Foundation Trust, Sheffield, United Kingdom

**Keywords:** postnatal depression, postpartum depression (PPD), stigma, motherhood, patriarchy, chemical imbalance, experience, narrative

## Abstract

**Background:** Despite affecting 15% of new mothers, experience of postnatal depression has often been hidden by stigma, cultural beliefs, and lack of medical understanding. We examined the barriers to women sharing their experience and gaining help, using their own words to illuminate the experiences of stigma and injustice. This study examines the narratives of women across the twentieth century, explores cultural movements that framed and contextualized their experiences, and marks how women became more empowered to speak of maternal distress.

**Methods:** Narrative literature was identified via searches of literature catalogs. Narrative accounts provided a lens through which to analyze cultural understandings of postnatal depression according to historical method. Contemporary medical and sociological literature discussing postnatal depression was used to contextualize the social climate within which these narratives were written. This work combines historical analysis with philosophical framework to develop insight into patient experiences of mental ill-health and associated stigma.

**Results:** This research identified three core cultural movements providing women with a framework in which to discuss their experiences of postnatal depression: the labor movement in the early twentieth century, the second-wave feminism movement in the mid-twentieth century (ca. 1960–1980), and the so-called “Prozac revolution” emerging at the end of the twentieth century. These movements provided distinct culturally acceptable etiologies around which women were able to frame their experience of postnatal depression. This provided women with space in which to share and process their experiences and aided them in overcoming contemporary stigma against mental illness by challenging disparaging stereotypes of the depressed mother.

**Conclusions:** Despite the stigmatizing nature of mental illness, women have demonstrated resilience and ingenuity by utilizing acceptable cultural movements to reframe their experiences of postnatal depression, challenging traditional perceptions of motherhood and effectively earned recognition of their sufferings. During this period, concordance between patient perceptions of postnatal depression and clinical understandings of the condition has been variable. Highlighting the detriment to therapeutic relationship when discordance is present, the narrators have demonstrated the need to destigmatize illness and facilitate cooperation between physician and patient and remind clinicians of the importance of placing patient experience at the center of care.

## Introduction

In this article, we will explore how women have written about their postnatal mental health over the last 100 years. A series of poignant, sometimes agonizing, narratives reveal the women who challenged societal stigma to share their experiences of postnatal mental illness in the twentieth and early twenty-first century. Utilizing theories of epistemic injustice identified by philosopher Miranda Fricker, this article also delineates how women have used cultural movements to reframe and contextualize their experiences. Presenting their experiences in a more accessible, and perhaps more acceptable, way has allowed women to communicate with a society that highly stigmatizes mental illness and frequently devalues female experience. As a piece of interdisciplinary research, this article combines historical analysis with philosophical framework to provide clinicians with greater insight into patient understandings of mental illness and experience of stigma.

In 1920, the notable British writer and sociopolitical activist Marie Stopes proclaimed emphatically that “Every lover desires a child,” thus introducing her latest work, *Radiant Motherhood: A Book for Those Who Are Creating the Future* (1). Her insistence was that parenthood, and motherhood particularly, was an integral feature of adult life and relationships was echoed by contemporaries, and it continues to be echoed by many in the society today. Ideas around female identity have long been intertwined with pronatal rhetoric, on both a societal and personal level (2). One doctor stated in 1911 that “[children] are as necessary to [a woman's] happiness as the food she eats and the air she breathes” (3), while actress Brooke Shields wrote in 2005 that she had “always dreamed of being a mommy” (4). Traditional narrative of motherhood has framed it as a time of excitement and affection, particularly in the first few fleeting months after delivery. However, with estimates suggesting that around 15% of women experience the mental health condition postnatal depression (5), it is evident that this narrative is at best an embellishment and at worst a falsification of the reality many women face in early motherhood.

Like many mental health conditions, postnatal depression has at times been stigmatized, poorly understood, and misrepresented. Today, as many as 58% of women experiencing postnatal depression will not seek help or speak out about their experiences, with many citing they were “too scared” to seek help (5). Furthermore, the ambiguity associated with the condition due to its perinatal onset has often caused it to be marginalized, an issue that neither falls entirely under the remit of psychiatry or women's health. Combining this marginalization with patriarchal traditions that have ignored women's voices, constituting what Miranda Fricker terms “testimonial injustice,” results in both historical research and qualitative psychiatric research having overlooked the condition frequently. There is no mention of the topic in otherwise-comprehensive histories of depression and psychiatry [such as that of (6–8)] and limited exploration in histories of obstetrics (9).

This pa*per seeks* to begin closing this gap in the academic literature by exploring the relationship between stigma and the cultural understandings of postnatal depressive illness throughout the twentieth century. The literature included is primarily British in origin; however, given the great cultural exchange between Britain and the United States and the dominance of American culture on the international stage, some works of American literature have also been included. Those included interact with, mirror, or are otherwise relevant to themes highlighted in contemporary British literature. They work, therefore, to complement the British dataset analyzed, rather than provide a comprehensive review of American attitudes toward postnatal mental illness. The article focuses on three periods of time in which key narrative texts have been identified. These are the early twentieth century (1910–1925), the early 1960s to the early 1980s, and the turn of the twenty-first century. These periods have been chosen, as they are central periods of discussion on postnatal depressive illness in which a noticeably higher volume of literature concerning postnatal depression was published. Furthermore, each period is dominated by distinct sets of ideas pertaining to postnatal mental illness, and jumping between these periods allows this article to effectively summarize the evolution of postnatal depression over the preceding 100 years.

## Methods

### Source Selection

Narrative accounts of postnatal depression constitute the primary literature analyzed in this article. These texts are used as a lens through which to view cultural understandings of postnatal depressive illness in the twentieth and early twenty-first century. Complementary texts, such as medical literature or literature of sociological and cultural importance, are also used to contextualize the social climate within which these narratives were written, better delineating the impact of societal stigma on experience of postnatal depression. All texts were written in the vernacular, in English.

The use of “illness narrative” in academic research, once referred to as an “orphan genre” by Arthur Frank, has enjoyed increasing popularity as a data source across both clinical disciplines and medical humanities in recent years (10). On the use of narrative in medical disciplines, Dr. Angela Woods has written:

“Advocates for the use of narrative have a commitment to understanding the centrality of the illness experience in the medical treatment of disease, taking seriously stories of illness, and valuing the individual as the empowered author-narrator of her own story” (11).

However, in the same piece, Woods highlights several limitations that she perceives with analysis of narrative. These include a tendency to “overlook the cultural and historical dimensions of narrative form” and “overinflating what counts as narrative…including painting, poetry and dance” (11). This article has addressed these concerns in its methodology: the former, by including in this analysis supplementary literature contemporary to the narrative accounts and the second in the selection process for inclusion in this work.

“Narrative” in this instance has been defined as first-person accounts, all of which describe the emotional experience and impact of mental distress resembling contemporary understandings of, or identified by the author as, postnatal depressive illness. The narratives chosen for inclusion were largely autobiographical letters, memoirs, and chapters. Also included were excerpts from published interviews or oral accounts of early motherhood because, like the autobiographical pieces, they provide first-person recollection of experiences.

To compile the literature used, a range of databases, both academic and commercial, were searched. Academic databases searched were the EBSCO Historical Abstracts database, Literature Online (Lion), and PubMed. The search terms used were one of “postnatal depression,” “postpartum depression,” and “motherhood” in conjunction with one of “history,” “memoir,” “narrative,” “autobiography,” “diary,” or “account.” These early literature searches focused on establishing the type of literature that had already been published examining postnatal depression from a historical perspective. Indeed, this search proved limited. A search of medical database PubMed, for instance, with the search terms “history” and “postnatal depression” revealed a plethora of studies examining trends among women with a medical history of postnatal depression but little in the realm of historical or qualitative research. The Lion and EBSCO searches were equally limited, indicating that little historical research on the phenomena of postnatal depression was available. However, examining the bibliographies of the few secondary sources identified in these searches proved useful in identifying primary sources of discussion on postnatal mental health.

Expanding the repertoire of databases, the historical archives of The Guardian and The Observer were also searched; when searching these archives, the search terms “motherhood,” “maternity,” “postnatal depression,” and “postpartum depression” were used to identify articles discussing postnatal mental health. This search yielded a multitude of confessional letters in which women discussed their experiences. It also provided wider societal context for discussion of postnatal mental health and motherhood. We also searched other databases such as Amazon Books and Google Books with the same search terms. While unconventional search engines in academic literature, not only did they yield a plethora of published works, but they also indicated which texts had been most popular in terms of sales and were particularly useful for identifying later (published 1990s−2000s) literature. In using articles published in the press, we recognize that narratives identified may not represent an impartial view of public opinion, prone as such outlets are to sensationalism. We do not claim to provide an unbiased survey of women experiencing postnatal depression in the twentieth century; however, we do believe the sources selected represent the tone and content of published literature exploring experiences of postnatal depression. The combination of search methods used above, while atypical in scientific research, constitutes sound historical method used in the humanities.

In addition to synthesizing a timeline of narrative, concurrent timelines of medical and sociological literature discussing motherhood and perinatal mental health were also created. The medical timeline was largely put together through analysis of contemporary textbooks, which provide a good basis for understanding the dominant views of the medical community at the time of publishing. Also included were articles from prominent British and American medical journals, notably, *The Lancet, The British Medical Journal, Journal of the Royal Society of Medicine, Journal of Mental Science*, and *Obstetrics & Gynecology*. The sociological literature included in this discussion focuses on that produced by prominent female writers, discussing mental health and motherhood from a feminist–sociological perspective. This group of literature includes some of the most influential and acclaimed feminist works of the twentieth century, such as Betty Friedan's *The Feminine Mystique*. When analyzed alongside their contemporary narrative texts, the exchange of ideas between these streams of literature is evident. However, given the abundance of sociological literature discussing mental health and/or motherhood, it must be acknowledged that concentration on this specific stream of sociological literature has overlooked wider sociological perspectives on motherhood and mental health. To include other streams of thought in sociological literature would have been beyond the scope of this research.

### Definition of Terms

The integrity of this article relies on sound definition of the terms used in this piece. As discussed above, narrative here has been strictly defined as first-person accounts of the emotional experience and impact of postnatal depression. However, it is essential that we also clarify what constitutes “postnatal depression” in this article.

Modern psychiatry textbooks will typically identify three forms of mental disturbance that commonly occur shortly after the birth of a child. The first, “postnatal depression,” usually receives the most attention in discussion (12). Clinical features associated with postnatal depression include emotional disturbances such as negative thought, low mood, anxiety, and feelings of guilt. It may also include physical symptoms such as trouble sleeping, tearfulness, and appetite changes. Particularly distressing are thoughts of harm either to oneself or to the baby. According to many health authorities, including the National Health Service (NHS), symptoms of postnatal depression must last for more than 2 weeks and typically occur 3–4 months after delivery, although they may appear at any time in the year following the birth of a child (12). The International Statistical Classification of Diseases and Related Problems (ICD-10) does not consider postnatal depression to be an entirely separate phenomenon from depressive illness occurring at other times in ones' life but does afford the condition distinct classification in recognition of the unique circumstances arising immediately after the birth of a child (13). The other two forms of perinatal mental disturbance, “baby blues” and “postpartum psychosis,” while sharing some features of postnatal depression, are considered separate phenomena and are beyond the scope of this research.

The definition of postnatal depression given above is corroborated by a number of British and American health authorities (12). However, this definition is a relatively recent development in the history of medicine, emerging in the late 1970s. The phenomena resembling the symptoms of postnatal depression have been described by a number of terms in the twentieth century, including, but not limited to, “puerperal insanity” (14), “puerperal melancholia” (15), “childbirth depression” (16), “postpartum emotional distress” (17), “depression with childbirth” (18), and “postpartum depression” (4). Additionally, the aforementioned “baby blues” has at times also been used interchangeably with “postnatal depression.” While the so-called “baby blues” share many features of postnatal depression, it is an experience now defined as transient in nature and not largely regarded as pathological. This article will not retrospectively apply the term “postnatal depression” to the literature published before this term became well-defined and its use widespread; however, care has been taken to ensure that literature included describes an experience of sustained distress comparable to modern understandings of postnatal depression. In particular, this applies to the narrative texts included in this research. Furthermore, to avoid misrepresenting the experiences of women who have not themselves identified their experiences as postnatal depression, we will refer to these experiences as episodes of “maternal distress.” This, we feel, as well as respecting historical context, also respects the attitudes of the women who wrote these narratives, who for numerous reasons may not have identified their experience according to contemporary medical labels.

### Analytic Techniques

These experiences collated will be analyzed in chronological order, with supporting contemporary academic works included in the analysis to contextualize them. Primarily, this article is an interdisciplinary work that uses historical perspective to analyze the relationship between cultural understandings, societal stigma, and etiology of perinatal mental illness through the voices of narrative authors. In addition to exploring and amplifying patient voice, this analysis has meaningfully and representatively charted the development of the condition we now understand as “postnatal depression” throughout the twentieth and early twenty-first century.

Also pertinent to this article is the phenomenon of epistemic injustice described by Fricker (19). Fricker describes epistemic injustice as an “umbrella concept,” in which an individual is “wronged in their capacity as a knower” (20). While the concept of epistemic injustice has been expanded by modern scholars, this article will focus on the application of Fricker's early denominations of epistemic injustice: the concepts of testimonial injustice and hermeneutical injustice. In addition to examining ideas articulated by the authors of the narrative pieces, this article will analyze how the phenomena of testimonial and hermeneutical injustice have been applicable to the experiences of the women studied, further developing the contextual understanding of the literature that these women have left for us. Incorporation of Fricker's philosophical ideas into the analysis of these narratives expands the scope of this article, encapsulating the interdisciplinary nature of the medical humanities.

## Results

As the primary source of data in the article is the narrative accounts of postnatal depression produced by sufferers themselves, this section serves to summarize these texts and review the main themes highlighted in these pieces. Concurrently, these pieces are contextualized and compared against wider contemporary literature. Analysis of the ideas highlighted by the narrators in these accounts of postnatal depression provides an understanding of how sufferers reconciled their experience with their own personal understanding of mental illness and societal stigma toward mental illness.

### Accounts of Motherhood in the Early Twentieth Century

A collection of letters compiled by Margaret Llewelyn Davies, secretary of the Women's Co-operative Guild (WCG), presents the maternity experiences of a group of working-class British women published in 1915 (21). The WCG was a faction of the cooperative movement focusing specifically on issues affecting working-class women. These anonymous letters formed part of a campaign headed by the WCG to provide financial assistance to pregnant women; the letters were shared with politicians to effect change to the current maternity welfare program. The women featured in these letters do not refer to experiences of distress with any contemporary nomenclature. However, they described feelings associated with both contemporary descriptions of “puerperal insanity” and modern understandings of postnatal depression.

One woman (referred to as Guildmember A in this piece) featured in Llewelyn Davies' *Maternity: Letters from Working-Women* (1915) described her emotional struggles following the birth of her third child:

“Many a time I have sat in daddy's big chair, a baby two and a half years at my back, one 16 months and one 1 month (sic) on my knees, and cried for very weariness and hopelessness. I fed them all as long as I could, but I was too harassed… The strain was fearful, and one night I felt I must sleep or die—I didn't much care which”—p. 45–46, Guildmember A, *Letters from Working-Women*, 1915.

Similarly, a second woman (Guildmember B) spoke of a “breakdown” following the birth of her second child and a feeling of having “very nearly lost all my spirit” (p. 140–141). Her language alludes to a hopelessness characteristic of depressive illness, such as feeling she “did not seem to have strength enough to drag through day after day” and having “felt like giving in altogether.” She also stated that during this time “life was a weary existence.” A third woman (Guildmember C) described a case of “nerves” (pp. 181–183), while a fourth (Guildmember D) described having isolated herself from others due to a “weakness” suffered after the birth of her first child (p. 43).

Common to all four of these women is an absence of medical terminology to describe their experiences, despite having relayed experiences resembling contemporary descriptions of puerperal insanity (22). In addition to avoiding the label “puerperal insanity” itself, other descriptors with medical connotation such as “depression” or “melancholia” were also avoided in most of the narrative accounts. This begs the question, why? Was this a conscious distancing of their experiences from mental illness, or did it arise from a lack of understanding of perinatal illness in the early twentieth century?

Also notable in this publication is the deep shame with which women spoke of their experiences of maternal distress. Guildmember D writes with apologetic tones, stating she “kept all to [herself] and was “ashamed to own up” to her experience of “weakness” following the birth of her first child. While she describes symptoms not dissimilar from those now associated with postnatal depression, she did not specifically refer to her mental state in her letter. The language she used attests to the shame she felt for her condition, the label “weakness” itself having connotations of inadequacy, feebleness, and personal shortcomings. Likewise, Guildmember C expressed that she “could have gotten advice” regarding her condition but refrained from doing so out of “fear” that “they would only laugh at me.” The shame evident in the accounts of these women is highly suggestive of a widespread stigmatization of mental distress in early motherhood.

Additionally, Guildmember A explored etiology in her account of maternal distress, claiming that “the root cause is lack of rest and economic strain—economic strain being the greatest factor for ill” (p. 46). This is significant, as it demonstrates that this writer was broadly aware of, and actively challenging, cultural understandings of mental illness (7). Prominent etiological models of the time, which will be explored further in later paragraphs of this article, considered mental illness as hereditary and therefore inevitable and incurable. The etiology also introduced classist undertones, as the hereditary causation led to middle- to upper-class society perceiving of the emergence of a “race” of “degenerate” lower class sufferers (23). Guildmember A's language suggests an awareness of this perception and is critical of the association of the lower classes with “degeneracy” and “feeble-mindedness,” stating that her living conditions would certainly be “enough to upset the mental balance of a Chancellor of the Exchequer.”

The predominant themes of narrative accounts in the early part of the twentieth century can be summarized as those of shame and uncertainty. The timidity with which women discuss their experiences, apologetic tones, and lack of engagement with sophisticated medical nomenclature is indicative of the stigma and shame associated with mental illness in the early twentieth century. The avoidance of medical labels may also intimate the lack of health literacy among these women. Moreover, there is evidence of discordance between the views of women experiencing maternal distress in the early twentieth century and those of the medical community, with sufferers highlighting the importance of environmental factors in the etiology of their experience. The externalization of causation highlighted by Guildmember A may represent an attempt to challenge or overcome the stigma she perceives in contemporary etiological theories.

### Degeneration and Depression: Maternal Mental Health 1910–1925

While we have seen that discussion of mental well-being after childbirth was a popular topic of discussion among the women of the WCG, medical literature published in the early twentieth century had lost its focus on postnatal mental illness. London-based physician Geoffrey Clarke noted in 1913 that “many of the more modern text-books do not devote even a short chapter to the so-called puerperal insanity” (14). This sentiment was also echoed in the United States (24). It is perhaps for this reason that the women writing of their experiences in *Maternity: Letters from Working-Women* avoided using medical nomenclature in their discussion—their contemporary doctors may not have recognized or considered their experiences as illness. Furthermore, as self-described working-class women, access to healthcare and health education was for these women was, at best, greatly limited. Early National Insurance did not extend to women who were not working. Guildmember A exemplified this when she described pleading for medical assistance despite her poor financial circumstances, asking “Doctor, I cannot afford you for myself, but will you come if I need?” (21).

The lack of health literacy among these women, and the omission of medical labels in their literature that may arise from this, is an example of what Fricker has termed “hermeneutical injustice.” Hermeneutical injustice occurs when the “shared resources for social interpretation” that allow one to make sense of one's experience are inadequate to describe one's experience (19). As described above, few women writing in *Maternity* possessed the resources to access healthcare when needed, and so it is not unreasonable to suggest that medical labels for their experience were simply beyond the vocabulary of some of these women. Furthermore, when describing mental illness, physicians themselves typically applied these labels to a different type of patient. Psychiatry in the early twentieth century was preoccupied with the institutionalized patient—it was not until the 1950s and 1960s that community psychiatry began to take hold in the United Kingdom (6). The descriptions of mental disturbance published in *Maternity*, while distressing and unsettling, had not resulted in asylum confinement. Such experiences would have flown under the radar of many contemporary physicians such as Clarke who studied institutionalized women. The women writing to the WCG in 1915 may, therefore, have omitted labels from their accounts owing to the hermeneutical injustice denying them the vocabulary and knowledge required to enable them to unify their experiences under a common banner.

However, analysis of wider attitudes toward mental ill-health, particularly during motherhood, elucidates other factors that contributed to this distancing of experience from medical labels, as evident in *Maternity*. Since the nineteenth century, influential psychiatrists such as Benedict Augustin Morel and Henry Maudsley had asserted that mental illness arose from an inherent “degeneration” and was therefore hereditary (23). Clarke in 1913 notes that “congenital mental defect was noted in a good many cases,” indicating that he too favored a hereditary etiology of mental illness. This etiological theory was widespread in the early twentieth century, with one physician in 1911 writing to the *Journal of Mental Science* “I take it for granted that we all agree that [heredity] has an enormous influence in the production of insanity” (25). These ideas were assimilated comfortably into a wider societal movement—that of eugenics. The eugenics movement was increasingly popular in the early twentieth century, and it used fear for the “quality” of future generations to try influence social attitudes, public health initiatives, and even the law (23).

Writing about distress in motherhood from a eugenicist standpoint were physician Elizabeth Sloan Chesser and academic campaigner Marie Carmichael Stopes. Sloan Chesser wrote of “nerve strain and anxiety” experienced by women in early motherhood (26), recognizing the difficulties faced by women such as those writing in *Maternity*. Similarly, Stopes described a period of “unbalanced mind” in the postpartum period, observing that in some women, “bearing of a child [results] in a weakening of the sub-conscious control over her emotions” (1). Both Sloan Chesser and Stopes felt that mental illness resulted from a hereditary predisposition, with Sloan Chesser asserting that “hereditary taint is the most predominant factor” (p. 202), while Stopes' attributed “degenerate, feeble-minded and unbalanced” traits to the “little understood force ‘heredity’” (p. 2).

However, elsewhere in her publication, Sloan Chesser also observed that “the burden of maternity under present conditions is a source of terrible hardship” and advocated for improvements to conditions such as housing and education to reduce the incidence of “mental exhaustion” (pp. 96–97), showing that Sloan Chesser was exploring multiple ideas and causes but fitting these into an overall eugenicist position. Therefore, Sloan Chesser's work presents a complicated picture of contemporary understandings of postnatal mental illness through which to evaluate the narrative of the WCG. On the one hand, she writes firmly that mental illness is a hereditary affliction, concurring with their male contemporaries, particularly Clarke and Faulkes. This creates an environment in which mental or emotional disturbance are considered stigmatizing and shameful occurrences, hallmarks of “degenerate” stock and therefore provides motivation for women to avoid labeling their experiences as such. On the other, Sloan Chesser also separates some forms of mental and emotional disturbance in the postnatal period from the traditional labels of mental illness.

Like Guildmember A, Sloan Chesser's work offers alternate etiology in the form of pressured living conditions. This suggests that while the stigmatizing hereditary etiology did dominate psychiatry at the time, there were efforts on both the part of the sufferers and academics to reframe the condition in a more favorable light. It also alludes an awareness on the part of Guildmember A, suggesting that she is going to great lengths to carefully frame her experience in an acceptable manner, within the wider context of the stigmatizing hereditary etiology. The concordance between a sufferer and academics demonstrated by Guildmember A and Sloan Chesser is unusual for the period. Stopes, on the other hand, makes a less sympathetic case for mental illness in working-class mothers. Interestingly, Stopes portrayed symptoms of mental distress in early motherhood as “not a thing to fear or be ashamed of” when they are exhibited by “a mother-to-be who deeply desires her child…living under comfortable, protected and happy conditions” (pp. 36–37). Conversely, according to Stopes, those “feeble-minded” mothers “living in the worst of slums” had emphatically fallen victim to the “little understood force ‘heredity’” (1). The classist distinction by Stopes is typical of academic literature at the time, which was heavily influenced by the eugenics movement. Indeed, we have seen earlier that Guildmember A sought to address these class distinctions in her narrative. In addition to describing how her living condition would be “enough to upset to the mental balance of a Chancellor of the Exchequer,” she deploys language that is often used by the upper classes that developed this etiology, stating that present maternity conditions will result in “race suicide.” This again challenges the then-popular medical and eugenicist notions that mental ill-health was the inevitable fate of the tainted, feeble-minded lower class.

A further clue to understanding the level of insight with which the women of the WCG were writing can be found in the writing of Guildmember C. She indicates in her narrative that she did view her experience as an episode of illness, as she sought medical advice (p. 183). Indeed, it was the doctor himself who made the diagnosis of “nerves” (21). This therefore suggests it was the narrator herself who skirted the medical terms, writing euphemistically of “bad times” and “suffering” instead of applying medical nomenclature to her experience. Whether the narrators of the WCG made conscious or unconscious language choices to avoid associating their experience with mental illness cannot be said, but it is evident that stigma had an enormous impact on their experience and is readily reflected in their writing.

### Revolutionary Accounts: Accounts of Motherhood 1960s−1980s

Writing in 1915, the women featured in the WCG's *Maternity: Letters From Working-Women* had no right to vote were barred from many professions and had never had representation in parliament. Moving forwards 50 years, the fight for women's rights had moved beyond suffrage to become “women's liberation.” This section will analyze some of the literature produced during the period widely associated with the Women's Liberation Movement, beginning in the 1960s and stretching into the early 1980s. The social and cultural changes of these years enabled the movement to carve out a new space within which postnatal mental illness could be discussed, and subsequently new forms of narrative emerged. The Women's Liberation Movement saw the growth of “consciousness raising” groups, which created spaces for women to come together and discuss their experience. Within these groups, women, supported by peers and comrades, began to share and value their experiences (27). Discourse from these groups spilled out into a wide range of literature; sociological works focused on female experience, candid descriptions of female experience circulated in popular newspapers, and a number of part-autobiography, part-investigative journalism critiques of motherhood were published.

Early in this period, two ground-breaking social studies on the experiences of women were published almost simultaneously, which independently identified a revolutionary new theme in discussion of perinatal mental distress. In 1963 and 1966, respectively, Betty Freidan and Hannah Gavron introduced the argument that the pervasive, patriarchal social norms that idolized motherhood were in fact a deception, a false dream that could only result in misery when women confronted the harsh realities of motherhood after the birth of their child (16).

Both Gavron and Friedan featured several extracts from the women they had interviewed, who recounted their realization that their expectations of motherhood had fallen far short of their experience. One woman confided that she felt “so empty somehow, useless” in her role as a housewife and mother (16). The language of the interviewee echoed Friedan's ideas; feeling “empty” in particular conveys the sense of hollowness and the lack of fulfillment found within the role of housewife–mother. Likewise, Gavron identified conflict between the vision of motherhood society sold to women and the realities they experienced, featuring a woman who expressed that she “felt such a failure not knowing whether the baby was warm enough, or fed enough, or why it was crying” (28). Naming herself as a “failure,” she intimates that she equated success with motherhood in the same way that the archetypal motherhood-as-fulfillment narrative presents it. She also apportioned the blame entirely on herself for struggling to cope with the labors of childcare, suggesting an expectation that child rearing was to be solely her responsibility. According to both Friedan and Gavron, the crisis of identity that resulted from the conflict between the archetypal “ideal” and the realities of motherhood resulted in great emotional distress for many new mothers.

Of course, there are methodological issues with narrative provided by work of this type—how far were Gavron and Friedan selective in their choices of what to include in their books? How representative were these particular experiences? However, it is clear that the archetype of motherhood-as-female-fulfillment—and the distress it caused when it failed to match reality—continued to be pervasive throughout the period, as the theme was revisited again in later narratives.

The autobiographical preface of American author Adrienne Rich's feminist critique of motherhood, *Of Woman Born: Motherhood as Experience and Institution* (1976), relayed a similar sense of distress and disenfranchisement in taking on the role of a mother for the first time. Rich described herself as “an anti-woman—something driven and without recourse to the normal and appealing consolations of love, motherhood, joy in others” (29). Identifying as the “antiwoman” when experiencing postnatal mental distress, Rich demonstrates the deep intertwining of motherhood with one's identity as a woman. Her experience is evidence of the identity struggle faced by new mothers as they tried to reconcile their feelings of distress and despair with the dreams of fulfillment and happiness that they had imagined. Given that Rich's work was strongly influenced by Friedan's initial critique of the role of the housewife-mother, it is perhaps unsurprising that Rich echoed the dissatisfaction with the false-promise of fulfillment and happiness in motherhood that she felt she was sold by society. Other subsequent work followed the same structure and echoed these themes, notably that by Oakley (3) and Welburn (30).

Into the 1980s, women continued to write on the theme of fulfillment in motherhood. As well as the semiautobiographical commentaries produces by Rich, Oakley, and others, “ordinary” women were also sharing their experiences of postnatal depression in national forums. One woman, Alison Coles, might be used as an illustrative example. She wrote to the *Guardian* in 1986 to question the image of motherhood that she had been sold all her life, asking “was this all there was?” (31). Again, Coles highlighted the discrepancy between the ideals of early motherhood and the realities of it. Coles did not come from any kind of academic background herself, but her narrative demonstrates that the feminist analysis and critique of motherhood had been internalized by women of the period and was being incorporated into their understandings and experiences of postnatal depression. In the same way that the WCG had been able to provide an outlet for women's experiences in the cooperative movement, women's liberation in the 1960s, 1970s, and 1980s enabled women to discuss their experiences and had provided a vocabulary with which to articulate them.

In addition to the disconnect between expectations and experiences, later narratives began to identify a second factor that was damaging women's participation with early motherhood. Maternity care had changed dramatically since the 1910s. While almost all births took place at home in the early part of the twentieth century, by 1960, approximately half of women delivered in hospital, rising to 98% of women by the early 1980s (32). In 1979, Oakley was one of the earliest to speak at length in her narrative of postnatal depression about how her experience of birth affected her subsequent mental health (3). Oakley spoke critically of her experience of childbirth, to which she attributed the difficulties she experienced bonding with her baby:

I remember myself as a passive patient, bewildered, afraid and alone, controlled rather than controlling, his birth more their achievement than mine. There was no euphoria, the baby in the cot was a threatening stranger… I was delivered of my own identity at the same time… it was a long time before I could remove the barrier of his birth from my relationship with him. (pp. vii–viii)

Also stating “the way in which a birth is managed could influence a woman's whole experience of being a mother” (p.v), it is evident that Oakley identified her experience of childbirth as a critical influence over her early experiences of motherhood. The move toward medicalized birth was a symbolic beginning to the helplessness, lack of autonomy, and relinquishing of control she experienced in early motherhood. For Oakley, it was also symbolic of the continued subjugation of women in British society. The medicalized birth, led by paternalistic, majority-male physicians, undermined women during a pivotal moment in their developing identity as a mother (3).

Writing to *The Guardian* in 1979, Ann Schofield also recounted an emotional “crisis” she experienced after the difficult birth of her second child. Schofield criticized the treatment she received from medical staff after a difficult, premature delivery (33). She described being told by a doctor “pull yourself together for your husband's sake” after her delivery. The doctor's language, appearing to lack in empathy toward Schofield, left her so “outraged” that she decided not to stay in hospital with her baby, who was in intensive care. However, this drove a further wedge between her and her baby, causing “anxieties” and “persistent and aggressive nightmares.” Schofield's distress continued, stating “it took me 2 years…to fully resolve the confusion of negative feelings associated with the birth.”

There is notable evolution in the discussion of postnatal depression in this part of the century when compared to the narratives produced in the 1910s. Mirroring the themes highlighted in the early narratives, undertones of shame and weakness are evident in both the language of the narrators themselves, who consider themselves as “empty” and “failures” and in the language of the clinicians interacting with them (“pull yourselves together”) (33). However, like Guildmember A in 1915, narrators have also highlighted alternative, externalizing etiologies for postnatal depression in order to overcome the shame and stigma they associated with their experience. Highlighting patriarchal social norms—the idolization of motherhood as a path to fulfillment for women, and the paternalistic, dehumanizing medical birth as means of undermining female identity—these narrators have reframed their experience in a way that allowed them to speak out and to challenge prevailing stigmas.

### Patriarchal Ideals and Postnatal: Maternal Mental Health in Society 1960s−1980s

Mirroring the women of the WCG earlier in the century, women writing in the 1960s, 1970s, and early 1980s had likewise found a cultural movement through which they could relay their experiences of postnatal depression. Like the women of the WCG, who utilized the cooperative movement's campaign for better living and working conditions to share their experiences, the Women's Liberation Movement had provided a structure within which mental ill-health could be more readily discussed. This movement highlighted the impact that strict societal expectations of women had on their well-being, which placed the onus of “fault” on society rather than personal failings. From here, women were able to share experiences of postnatal depression on the premise that it originated in problematic patriarchal identity constructs or their mistreatment by the male-dominated medical field. While many of the women writing on this theme had sociological backgrounds, it should be noted that their ideas were not largely integrated into medical discussion of postnatal depression until later in the twentieth century, despite sociology's increasing influence on other areas of psychiatry. Indeed, etiology of postnatal depression was poorly defined within medical communities in the 1960s and 1970s Britain (22). The space, however, that feminist narrators created within which they could discuss their experience remained fringe. This discrepancy was commented on by Welburn, who noted that it in medicine, “men must act, control, perform” (pp. 65–67) and could not allow women to occupy space within their field (30). There is evidence that the arguments put forth by these women remained marginalized, and experiences of postnatal depression remained stigmatized. We have seen how Schofield was admonished by her physician and told to “to pull herself together.” An equally admonishing reply to Schofield's account published the following week considered that Schofield's letter “oozed self-pity” (34).

There is further evidence of the conflict between narrators and the medical community during this period. It is clear that the experience of birth itself had become a major theme in narratives of postnatal depression by the end of the 1970s, but scientific literature had not yet caught up with this. Welburn criticized the medical profession for their lack of response to the distress that was being caused by this relatively new, highly medicalized approach to birth. The British Medical Journal, Welburn claimed, had refused to publish a letter from a doctor who had written that “dangers arising from accelerated labor are interference with the mutual attachment of mother and child and damage to the mother's confidence in herself as a mother and as a woman” (30). This opinion echoed the thoughts of Oakley and Schofield, who felt that their birth experiences interrupted their ability to bond with their baby and compromised their identities as mothers (3, 33). While the doctor who authored this letter demonstrates that not all in the medical profession were entirely ignorant of these concerns, Welburn argued that the BMJ's decision not to publish this letter were demonstrative of the wider views of the medical community. While anecdotal evidence that suggested a link between delivery experience and postnatal depression was beginning to emerge, no large-scale research had yet tried to establish an empirical link, despite the growing number of mothers who “blame [postnatal depression] on the childbirth, the whole thing” (3). Indeed, modern literature on the subject remains indecisive. A 2017 meta-analysis on the subject found cautiously in favor of an association between cesarean section and postnatal depression, although acknowledged that the association remains controversial (35).

The gradual emergence of medical literature in the late twentieth century, which, to some extent, appears to corroborate the anecdotal evidence first emerging in the 1960s, is evidence of a second type of epistemic injustice encountered by the authors of our narratives: testimonial injustice. Testimonial injustice is described as occurring when a hearer's prejudice causes the hearer to “give a deflated level of credibility to a speaker's word” (20). The BMJ, for example, appear to exhibit testimonial injustice against the women and patients informing the work of Dr. Bardon, the doctor whose work, according to Welburn, was rejected by medicalized-birth endorsing BMJ.

Additionally, the advent of “postnatal depression” (or approximate synonyms) as an *acceptable* label for women to identify their experiences emerged in discussion of postnatal mental disturbance in 1960s, 1970s, and 1980s. This provided women with the means to consolidate their experience in a way in which women writing to the WCG had been unable to. Indeed, in her own work Fricker uses the discussion of postnatal depression in the “consciousness raising” groups America's women's liberation movement as an example of tools that have been utilized to address hermeneutical injustice endured by women in Western society (20). This phenomenon is described as a “lifting” of “hermeneutical darkness” as discussion of postnatal depression expanded in the 1960s, 1970s, and 1980s.

Narrative in this period, while demonstrating a significant shift in the frameworks used by women to discuss their experiences of postnatal depression, continues to be hindered by societal stigma toward the condition. The backlash faced by narrators such as Schofield illustrate the persistent societal view of mental illness as a failing or form of weakness, comparable to the “degenerate” or “feeble-minded” sufferers of the 1910s. Likewise, the narrators of this period have mimicked the women of the WCG in finding a way to externalize etiology of postnatal depression, in an effort to overcome this stigma. As seen in the previous decade, the etiology they propose is largely rejected or ignored by medical authorities. Similarly, language associated with shortcoming such as “failure” continues to be employed by narrators, again reiterating the shame with which they endured their experience. There is, however, also evidence of a larger push-back against stigma in this period. For one, the volume of narrative literature produced in this period appears much higher. Additionally, the position of the narrator themselves has evolved. Where the working-class women of the WCG relied upon the collective power of the cooperative movement to share their experiences, women in the 1960s, 1970s, and 1980s were using a range of channels to share their stories. Space had been carved out in the academic literature, while popular newspapers were beginning to open their platform to the experiences of “ordinary,” individual women.

### Modern Mothers: Narrative at the Turn of the Century

Around the turn of the century, another cultural shift in the discussion of mental illness occurred. The 1990s and early 2000s oversaw a radical change in medical models used to describe postnatal depression. While historian Clarke Lawlor described psychiatry in the 1970s as “a mess of theories and practices that had little or no consensus” (p. 160), the end of the century brought about a radical new model for describing depression (8). The advent of Prozac, an antidepressant drug, revolutionized models of depression for medical professionals and lay-people alike.

Prozac (generic name: fluoxetine) was part of a class of drugs collectively known as selective serotonin reuptake inhibitors (SSRIs), which work by increasing the levels of a neurotransmitter, serotonin, available to neurons in the brain. This increase was linked to mood regulation and feelings of well-being. Several other types of SSRI would also be released in the late twentieth and early twenty-first century, although Prozac was one of the most popular. Prior to the advent of SSRIs, other psychotropic medications had been developed to treat depression-like symptoms, such as benzodiazepines (like Valium) or barbiturates. However, SSRIs stood apart from their predecessors with a unique selling point that would transform understandings of depression; while other medicines provided symptomatic relief, SSRIs professed to address the cause of depression, the so-called “chemical imbalance” of serotonin. Prozac excelled in clinical trials and when it hit European markets in 1987, its efficacy along with aggressive marketing campaigns lead to prescriptions for fluoxetine rising rapidly throughout the 1990s and 2000s.

This revolution enabled a new form of narrative on postnatal depression to arise. Lauded by some as “astonishingly honest,” “brave,” and “giving hope to countless women” (4), the early 2000s heralded the emergence of the celebrity exposé memoir. Perhaps, the most well-known of these was actress Brooke Shields' 2005 *Down Came the Rain*, although it was preceded by Marie Osmond's 2002 *Behind the Smile: My Journey Out of Postpartum Depression*. Shields' book became a bestseller, and to this day, she continues to be commended as an advocate for postnatal mental health, revered by some as the “poster girl” for postnatal depression (36). Further memoirs (albeit, less high profile) were also published by Kleiman (37) and British authors Aiken (38) and Busby (22).

Throughout the period, we see the language of “chemical imbalance” that had resulted from the “Prozac Revolution” becoming integrated into the narratives of women relaying their experiences of postnatal depression. For example, a 1992 article published in *The Observer* featured the case of “successful” modern woman “Jane.” Her intense experience of postnatal depression following the delivery of her first child left her feeling so trapped by helplessness that the “only thing was to kill myself” (39). However, Jane's remarkable recovery left her confident in the belief that her experience was “a chemical process.” Developing this further, she stated “that this is real and the answer is not the stiff upper lip.” Jane's confident assertion here demonstrates of the power of the idea of “chemical imbalance” (39).

This attitude is replicated again and again throughout the late twentieth and early twenty-first century. Like Jane, Kleiman also highlights the model of chemical imbalance—Kleiman explained that postnatal depression is “not something you brought on yourself” (37). Additionally, Shields directly and repeatedly reaffirmed the “chemical imbalance” model of understanding postnatal depression in her memoir (4). Like Jane and Kleiman, Shields utilized this model as a defense against the stigma associated with perinatal mental disorder, explaining that “in a strange way, it was comforting when my obstetrician told me that my feelings of extreme despair and my suicidal thoughts were directly tied to a biochemical shift in my body” (40).

These narratives indicate that this new model was welcomed by many women as a destigmatizing explanation of their experience, which legitimized the way they had felt, and, for many, also offered a reliable route to recovery. However, this is not to say that biochemical theories became the only explanation of postnatal depression. Kleiman also emphasized the importance of the “individualistic” approach to mental health, which was more reminiscent of sociological theories, imploring that “no woman is ‘just’ a disease, or just a chemical imbalance” (37). Additionally, both Shields and Busby regard their experience of childbirth as integral to their early experience of motherhood and the subsequent distress they felt in this role, echoing Oakley's work in 1979. Writing retrospectively in 2004, Busby explored the potential causes for her experience of postnatal depression in the 1980s. She paid close attention to the impact that delivery of her child had on her ongoing relationship with motherhood. Busby recounted the bewilderment and lack of control she felt during her delivery. Like Oakley in 1979, Busby too felt she was a passive onlooker in her own delivery, stating “I [hadn't] realized that C-Section stood for cesarean…I can honestly say that I had absolutely no idea what the obstetrician was talking about” (22). She also described feeling objectified by medical staff, reduced simply to a medical condition and not treated as an individual. “Do you mind” medical students asked, “only it's probably the only time [I] will get to see a transverse lie and CPD?” Busby felt this experience was belittling and isolating.

Shields had a similarly difficult experience during the birth of her daughter, delivered in 2003. While Shields recounts a better relationship with the medical staff present at her delivery, who she recalls treating her “gently” (p. 35), the experience remained overwhelmingly negative (4). Despite their care, she struggled to come to terms with having needed a C-section to safely deliver her baby, an experience that she felt rendered her “emotionally distant” from motherhood (p. 37). Most distressingly for Shields, she interpreted having not delivered vaginally as “a sign of my weakness and failure as a mother” (p. 86). The feelings of failure continued to haunt Shields throughout her experience of postnatal depression. Furthermore, she believed that her family shared this same judgement of her mothering capabilities, highlighting a perceived “disappointment” in the faces of her family when it was decided she should deliver through C-section. Another way in which Shields felt the C-section had contributed to her experience of postnatal depression was the sheer exhaustion and debilitating immobility she was faced with during recovery from surgery. The concentration of her energy onto recovering detracted from her focus on being what she considered a good, successful mother.

Another theme revisited in the narrative of turn-of-the century narrators Aiken and Shields was the idea that their lived experience of early motherhood had failed to meet to the expectations they felt that society had sold them. Echoing the arguments made by Freidan and Rich in the 1960s and 1970s, Aiken stated that “the ante-natal clinic had boosted up motherhood to such an extent. No one had told me the *truth*” (38). Shields, similarly, had placed great importance on becoming a mother as part of her own personal fulfillment. Her memoir opens with a short preface conveying her longing for motherhood.

*Once upon a time, there was a little girl who dreamed of being a mommy. She wanted, more than anything, to have a child and knew her dream would come true one day. She would sit for hours thinking up names to call her baby* (40).

The child-centered, fairy-tale language Shields adopts demonstrates the idealized version of motherhood she had envisioned. The presentation of this vignette at the very beginning of her memoir further emphasizes her dreams of motherhood. However, like Aiken, Shields found herself disappointed by the reality of her experience, asking “where was the bliss? Where was the happiness that I had expected to feel by becoming a mother?” (4). The recurrence of this theme across four decades is striking, and made more so by its prevalence in a variety of literary forms throughout this time. While criticism of the societal motherhood-as-fulfillment notion among feminist narrative is not unexpected, its appearance in celebrity memoir, and lay-person letters to *The Guardian* exemplifies how central many women find this theme to their experience of postnatal depression.

In particular, this theme was evident in the work of Professor Paula Nicolson, a psychologist in whose work the influence of early feminist literature was particularly evident. Her study of 24 British women's maternity experiences reiterated many of the arguments made earlier in the century; she felt that “romanticization of motherhood [that was] dictated by patriarchal power relations. It suits men for women to mother” (41). Nicolson continued to argue that society's archetypes and expectations of motherhood were responsible for the suffering of many women in the postnatal period—“all mothers are destined to disappoint themselves and their children” (p. 9). Furthermore, she felt that in the women she had interviewed, these factors were more prominent in their experience than the now-popular “chemical imbalance” model, stating that “despite cultural prevalence of the medical model in Western societies, most people who experience depression spontaneously provide an explanation” (42). Interestingly, there is evidence of consensus on this issue between women producing narratives and medical authorities in the early 2000s, contrasting the relationship between medical authority and feminist scholar evident earlier in the century. A leading psychiatric textbook published in 2005, for instance, recognized that for some women, “the hard work involved in the care of the baby” may be a significant causative factor in occurrence of postnatal depression (43).

The transition into the twenty-first century coincided with diverse discussion of postnatal depression in the narratives explored here. The detailed memoirs published by twenty-first century authors Shields, Aiken, and Busby allowed for deeper exploration of the experience of postnatal depression than the shorter earlier narratives had allowed. These intimate memoirs drew wider attention to the topic, with Shields being particularly notable for her contribution to public discussion of perinatal mental health due to her celebrity status (4). The advent of SSRIs and the subsequent “chemical imbalance” model also provided an opportunity for women to discuss their experiences. As several women intimated in their narrative, SSRIs provided a destigmatizing explanation for many women to utilize in their discussion of postnatal depression. Nevertheless, etiological models introduced by feminist–sociologist theory of the 1960s and 1970s, such as issues with the traditional societal perceptions of motherhood, continued to feature heavily in narrative of women experiencing postnatal depression. The diversifying of themes within narrative, combined with the intimate and comprehensive narratives provided by the memoirs published on the subject, demonstrated the way in which the destigmatizing of postnatal depression had allowed for the expansion of discourse on the topic.

### The Prozac Revolution: Postnatal Depression in Turn of the Century Society

As discussed above, the late twentieth century introduced a radically different etiological model to describe postnatal depression. The narratives demonstrate the extensive acceptability of this model; for the first time, a model developed by the medical profession was warmly received by the individuals the model purported to describe. While this etiological model internalized causation of postnatal depression by correlating it to “chemical imbalance” inside the sufferer's brain, this clinical, scientific explanation for the condition was a far cry from the accusatory “degeneracy” associated with a heritable etiology. Furthermore, this model was accompanied by a reliable method of treatment, demonstrating reversibility of the condition. In the same way that the WCG had associated mental distress with poverty and the feminist–sociological theories of the 1960s−1980s had externalized causation of postnatal depression in order to fight against the stigmas associated with mental illness, the “chemical imbalance” model had allowed narrators to separate the root of their distress from their own personal character.

We can also see how the relationship between medical expert and patient has moved on between the latter two periods discussed in this article. The experiences of Shields and Busby provide a good case comparison. Although delivering 30 years apart, both women delivered by emergency cesarean section and explore this experience in depth in their discussion of postnatal depression. There are notable differences between their experience; where Busby felt belittled and objectified by the attending medical staff (22), Shields lauded her obstetrician as “nurturing” (4). Indeed, the issues faced by Busby, highlighted by women such as Oakley and Coles in preceding decades, had been acknowledged by two successive government reports in the 1990s, The Winterton Report (1992) and The Cumberlege Report (1993) (32). Both reports advocated for choice and involvement of women in the delivery of their baby and crucially “the right for women to have control over their own body at all stages of pregnancy and birth” (32).

However, despite the apparent reconciliation between medical doctrine and patient experience emerging at the end of the twentieth century, narrative continues to acknowledge the themes first highlighted in the early 1960s. This is perhaps a testament to the power of these ideas, with Freidan and Gavron's critique of traditional female archetypes continuing to resonate with women such as Shields more than 40 years after they were first suggested. Indeed, the permeation of these ideas into the society is evident when we examine the backgrounds of the narrators who have relayed them. The journey of these ideas from their origins as highbrow, academic theories postulated by university scholars, to their incorporation into a lay-person's short media article, to their centrality in an enormous celebrity to memoir is remarkable.

Also remarkable in the work of turn-of-the-century narrators is the structural differences in the literature they produced. Notable to Kleiman and Aiken's work is a collaboration with physicians, who provided self-help style advice to new mothers experiencing postnatal depression. This is, of course, in stark contrast to the attitudes toward contemporary physicians relayed by the narrators in *Maternity* (1915) or later by Welburn and Oakley. This collaboration again reiterates the importance of acceptability when describing the etiology of mental illness and was perhaps made possible by the advent of the less stigmatizing, more favorable explanation the “chemical imbalance” theory offered to women.

## Discussion

### Limitations of This Work

While this article has endeavored to accurately and fairly represent the experiences of the women whose narratives are central to its development, experience is in its very nature personal and unique to the individual living it. Such work, therefore, cannot claim to present more than one researcher's interpretation of these texts. Interpretation is always subjective and vulnerable to the biases of the reader, unconscious or otherwise, and thus, it must be acknowledged that the arguments set forth in this paper are one of many sets of conclusions that may be drawn from the reading of this literature.

A further limitation to this work arises from the wide period covered by this article, with almost a century separating the earliest sources from the most recent. To progress with clarity through the twentieth century, I have focused this work on specific periods of time in which markedly distinct ideas around postnatal depression lead discussion of the topic. Naturally, this forces the research to skim over the interim developments in cultural understandings of postnatal depression. Furthermore, as stated earlier, the sociological aspects included in this research focused firmly on those postulated by a specific school of sociological thought, the feminist–sociological movement. We maintain that, given the vast interface between those ideas and narrative accounts, both produced by this movement and after it, this was an appropriate and fair representation of postnatal depression in British culture. However, we acknowledge the breadth of contribution that other fields of sociological study have made to modern understandings of postnatal depression.

When covering a significant period of time, it is essential to recognize that, in the interests of simplicity and succinctness, generalizations will be made. Unfortunately, it is often beneath these great generalizations that the nuance and detail that makes each and every story so extraordinary lies. In the face of such challenges, researchers must strive to balance the necessity of this practice with the duty to present the remarkable stories of twentieth century women with the sensitivity and intimacy they are owed.

This article has focused on the interplay between societal stigma toward postnatal depression and narrative of women experiencing the condition. This is just one aspect from which these works can be examined; there is still much to be learned from these exceptional insights into the difficulties of early motherhood, and further thematic analysis, such as examining the thought content or analysis of interpersonal relations in these narratives, would further enhance our understanding of the experience of postnatal depression. Similarly, in concentrating on the impact that etiology had on narrative, there remains space to chronicle in more detail the development of the condition we now understand as “postnatal depression” through focused analysis of the medical discussions of perinatal mental health in the twentieth century.

## Conclusion

This research has tracked the topics of discussion highlighted by narratives of postnatal depression in the twentieth century and evaluated the impact of societal stigma on the tools employed by narrators to share their experiences. In doing so, it has elucidated fundamental changes that have occurred in both medical and cultural understandings of postnatal mental illness. Throughout this changing landscape, women themselves have used cultural ideas to share and convey their distress in ways that were acceptable to contemporaries. By focusing on three distinct periods, this research demonstrates the development of postnatal depression throughout the century.

In the early part of the twentieth century, narrative was dominated by apologetic, shameful tones, while interpretation of the experience by the narrators differed vastly from the etiological models employed by the medical community to present postnatal mental illness. Women in the early part of the century also failed to label their experiences according to any contemporary medical terms. This research argues that this distancing of their experience from both medical terminology and the medical community's proposed etiology meant women were able to relay their experiences in more culturally acceptable ways. The cooperative movement provided a platform through which women's distresses could be heard.

Later in the century, the rise of the Women's Liberation Movement carved out another acceptable space in which to discuss postnatal depressive illness. While the movement criticized society from a feminist viewpoint, it mirrored the cooperative movement by creating an external, environmental basis from which postnatal depression arose. Despite the permeation of these ideas from academic literature into wider forms of media over the two decades associated with the Women's Liberation Movement, the ideas postulated in this movement remained fringe and were, in some cases, entirely rejected by the mainstream medical community. Thus, the disconnect between the medical community and the experiences that women described continued, and the stigmatism of these narratives persisted.

By the end of the twentieth century, advances in psychiatric pharmacology had transformed understandings of mental ill-health, both within the medical profession and in wider society. The release of SSRI antidepressant drugs propelled the “chemical imbalance” model of depression into public consciousness, where it was readily incorporated into patient narrative. The acceptability of this model allowed a consensus to develop between women experiencing postnatal depression and the medical profession—this was evident not just in women's own language but also by their collaboration with medical professionals when producing their literature. The acceptability of this medical model is evident in narratives produced at the end of the twentieth century and in the early twenty-first century. However, while the scientific approach to postnatal depression gave credence to the experiences of many women, narrative continued to highlight the feminist themes prominent in the literature produced in 1960s and 1970s. While the “chemical imbalance” model, then, had on the one hand helped created space in which women could talk about their experience, it had done little to address the engrained societal expectations of gender and how these affect ones' relationship with motherhood, as evidenced by the repetition of these themes over 40 years later.

In summary, two conclusions can be drawn from this research. First, that despite often stigmatizing cultural and medical attitudes toward mental ill-health, women have found outlets through which to share and discuss their experiences. Beginning with the cooperative movement in the 1910s, then as part of women's liberation in the 1960s, 1970s, and early 1980s, and finally by capitalizing on the end of the century's “Prozac revolution,” women have continued to demand that their voices be heard. Second, although being very much oppositional at the start of the twentieth century, there has been a movement toward concordance of ideas between pharmacology, the medical community, and the patient community that they ultimately endeavor to serve.

It may now be beneficial to ask whether or not women's voices have been heard if there were not scientific theories to describe their experiences. While work on serotonin provided a framing for discussions of postnatal depression, this framing was, of course, scientific, rather than narrative or experiential. It must be wondered whether had scientific framework had not emerged, we might still be disregarding women's narratives—and what this implication has on how we view experiential, narrative evidence in other medical arenas today.

## Author Contributions

The literature search and primary analysis of this research was done by GE. This was supplemented by the direction of CM and IS, whose detailed knowledge of medical history, philosophical frameworks, and medical humanities methodology were instrumental in drawing the conclusions from this work. IS was also central to numerous revisions of this research allowing for close analysis of the texts examined. All authors contributed to the article and approved the submitted version.

## Conflict of Interest

The authors declare that the research was conducted in the absence of any commercial or financial relationships that could be construed as a potential conflict of interest.
